# Ugi Four-Component Reactions Using Alternative Reactants

**DOI:** 10.3390/molecules28041642

**Published:** 2023-02-08

**Authors:** Seyyed Emad Hooshmand, Wei Zhang

**Affiliations:** 1 Department of Chemistry, Faculty of Physics and Chemistry, Alzahra University, Tehran 1993893973, Iran; 2 Department of Chemistry, University of Massachusetts Boston, 100 Morrissey Boulevard, Boston, MA 02125, USA

**Keywords:** multicomponent reactions, Ugi reaction, isocyanide chemistry, diversity-oriented synthesis, alternative reactants, rational design, green synthesis

## Abstract

The Ugi four-component reaction (Ugi-4CR) undoubtedly is the most prominent multicomponent reaction (MCRs) that has sparked organic chemists’ interest in the field. It has been widely used in the synthesis of diverse heterocycle molecules such as potential drugs, natural product analogs, pseudo peptides, macrocycles, and functional materials. The Ugi-4CRs involve the use of an amine, an aldehyde or ketone, an isocyanide, and a carboxylic acid to produce an α-acetamido carboxamide derivative, which has significantly advanced the field of isocyanide-based MCRs. The so-called intermediate nitrilium ion could be trapped by a nucleophile such as azide, *N*-hydroxyphthalimide, thiol, saccharin, phenol, water, and hydrogen sulfide instead of the original carboxylic acid to allow for a wide variety of Ugi-type reactions to occur.β In addition to isocyanide, there are alternative reagents for the other three components: amine, isocyanide, and aldehyde or ketone. All these alternative components render the Ugi reaction an aptly diversity-oriented synthesis of a myriad of biologically active molecules and complex scaffolds. Consequently, this review will delve deeper into alternative components used in the Ugi MCRs, particularly over the past ten years.

## 1. Introduction

The advent of multicomponent reactions (MCRs) is a promising platform for the synthesis of organic molecules, and it has been explored via the investigation of a high variety of components within a reagent class, as well as a growing number of components for the direct synthesis of a broad spectrum of chemicals [1,2,3,4]. MCRs, which use three or more accessible starting reactants in a single vessel, are an important class of sustainable and green synthesis, as they enhance orthodox “stop-and-go” synthesis, which requires tedious workup, isolation, and purification. In terms of green chemistry criteria, MCRs enjoy high atom, time and energy economies, a favorable E-factor, work under environmentally benign conditions and, importantly, reduce the amount of waste and/or byproducts [5]. Numerous MCRs, such as Strecker, Hantzsch, Bignelli, Mannich, Betti, Passerini, Gewald, Kabachnik–Fields, Asinger, Huisgen, Debus-Radziszewski, Povarov and Petasis, Groebke–Blackburn–Bienaymé have been developed and placed in the chemist’s toolbox, and immense unnamed MCRs have been developed and reported in recent years [6,7]. In spite of the central significance of serendipity in the discovery of MCRs, such as the Passerini’s three-component reaction, such observations play a far lesser part in efforts to afford MCRs, where rational design based on prior knowledge plays a much larger role. With the development of rational, well-defined reaction mechanisms, it is now possible to rationally design highly efficient new MCRs [8]. Of course, it should not be forgotten that the exact ratio of serendipity to creativity and rational design in the history of the discovery of some classic MCRs is not crystal clear [9].

**Figure 1:**
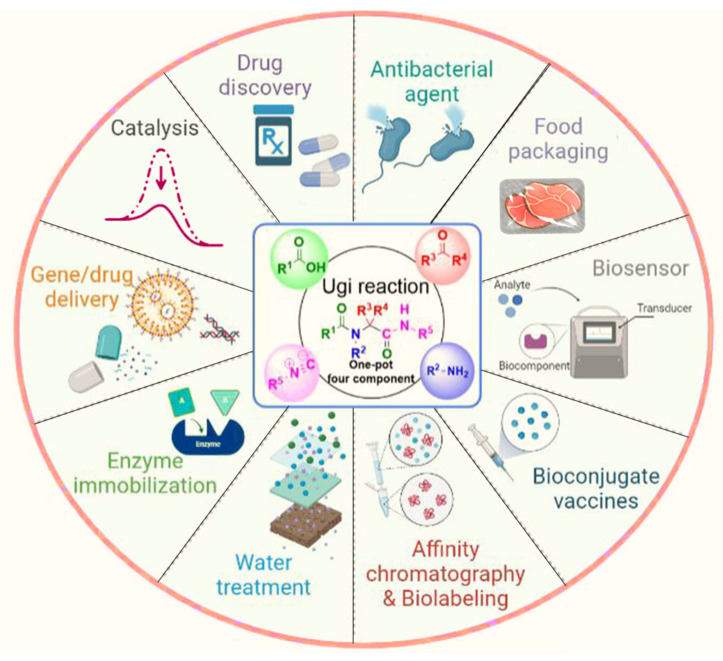
Applications of the Ugi-4CR in various fields of cutting-edge technology.

The Ugi-4CR, which was discovered in 1959 by the Estonian-born German chemist Ivar Karl Ugi, is undoubtedly one of the most utilized MCRs. In addition, a series of papers by Ugi in 1960s are recognized as the origin of MCR chemistry, which is good for library generation of screening sets [10] to produce products with a multitude of pharmacologically important properties [11]. In the Ugi-4CR, an amine, an aldehyde or ketone, a carboxylic acid, and an isocyanide are used to render α-acetoamido carboxamide derivative. Ugi MCRs have been broadly implemented in the direct synthesis of a vast number of heterocyclic rings [12,13,14], asymmetric scaffolds [15], biologically active molecules [16,17], and facile generation of macrocycles [18] and polymers [19]. The Ugi-4CR is taken into account as a crucial step in the production of various natural products [20]; for instance, the naturally potent α-amino acid antibiotic (+)-furanomycin II was prepared to utilize the Ugi-4CR [21]. In addition, many years of research investigation on this MCR have exhilaratingly culminated in the discovery of novel and effective synthetic methods for renowned drugs [22,23]. Ugi’s synthesis of the antibiotic penicillin through a highly convergent method is a beneficial example. Merck’s original process for the HIV drug indinavir (Crixivan) was an expensive and ineffective multistep synthesis. Incorporating a Ugi-4CR as a crucial step in the production of Crixivan decreased the number of steps, improved yields, and excluded the need for a laborious work-up or separation phase [24]. In another example, a best-selling medicine atorvastatin could be assembled through Ugi-4CR in an overall short and high-yield synthesis that performs better than the Paal–Knorr synthetic strategy [25]. To expand the repertoire of deploying Ugi reactions in modern technology, this worthwhile MCR has been applied in the field of materials functionalization [26,27] and used in enzyme immobilization [28], catalysis [29], water treatment [30], biosensors, gene/drug delivery [31], ligation and bioconjugation [32], and antibacterial self-healing hydrogel [33]. Interestingly, MCRs for productive vaccines [34] have been developed (Figure 1).

**Figure 2:**
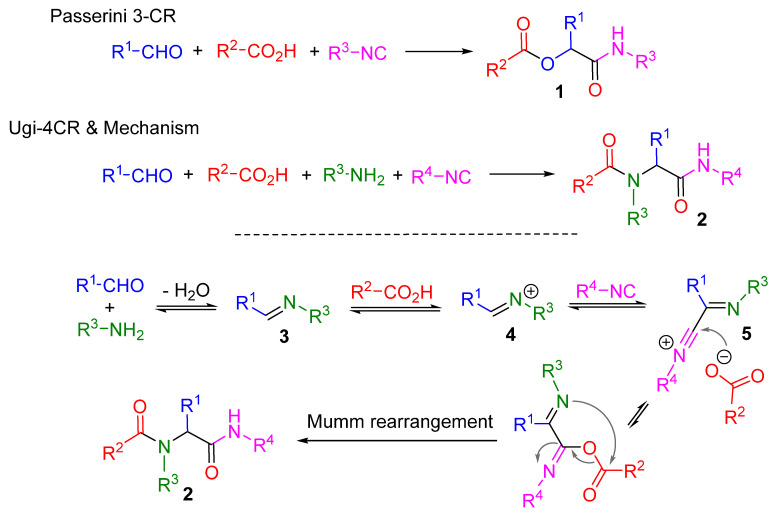
Classic Passerin-3CR as well as Ugi-4CR and its proposed mechanism.

In this context, the crucial question is whether Ugi reactions lie in serendipitous discovery or rational design based on Ugi creativity. The discovery of the Ugi-4CR seems to have been predicated on Ugi’s previous scientific understanding of the Passerini three-component synthesis of α-acylated α-hydroxyamides **1** utilizing the single reactant replacement (SRR) approach. The SRR methodology enables the extensive creation of new MCRs. Ganem coined the term SRR, which refers to the improvement of novel MCRs by systematic evaluation of the mechanistic or functional role of each reactant in a famous and well-known MCR [35]. In particular, Ugi was able to replace the carbonyl component used in the Passerini 3-CR with an imine, resulting in the renowned Ugi-4CR (α-acylaminocarboxamide **2**). As a result, with four diverse components and a variety of readily accessible starting materials, the Ugi reaction is recognized as a valuable and flexible synthetic method for the diversity-oriented synthesis of a significant number of organic molecules. The Ugi-4CR directly produces peptide-like molecules referred to as peptomers, diamide, or bis-amides, which are the peptidomimetics structures **2** [36]. The Ugi reaction has also dominated research efforts in both the search for new MCRs and the consideration of new scaffold diversity through post-MCR transformations and unions of MCRs [37,38,39]. Shown in Figure 2 are the classic Passerini-3CR reaction as well as Ugi-4CR. Regarding the mechanism of Ugi-4CR, it is proposed that an initial condensation of an aldehyde or ketone with an amine gives imine **3**, which is then protonated by an acid to form an iminium ion **5**. The latter is attacked by the nitrilium ion **4**, which produces isocyanide. Then, through a Mumm rearrangement from the precursor, carboxylate is added to the produced intermediate to produce the Ugi-4CR product **2** (the backbone of the bis-amides) [40].

In addition to four common components, there are several alternative reagents to these main components through which organic chemists employed to prepare molecules with elevated diversity and complexity and even as active pharmaceutical ingredients (APIs) [41]. We were thus enthused to review the area of the Ugi-4CRs and their alternative components in light of the significance of this MCR in the straightforward and facile synthesis of biologically active compounds and complex scaffolds.

## 2. Alternative Components for Carboxylic Acids

**Scheme 1:**
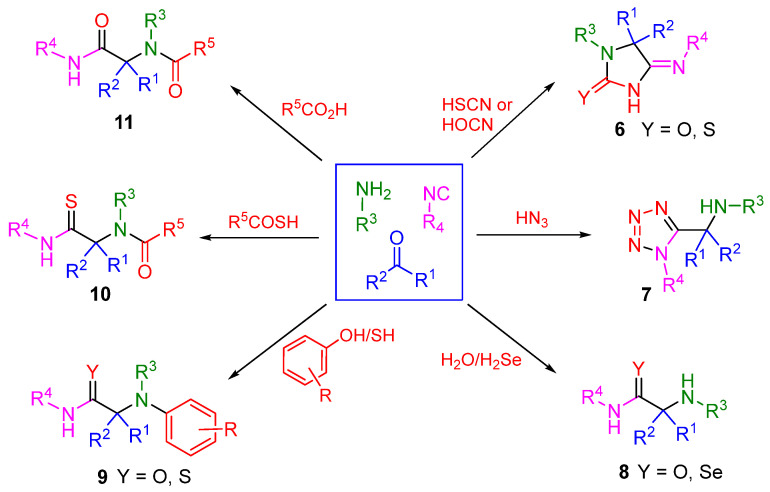
Use of carboxylic acid alternatives in the Ugi-4CR.

The ‘collection’ of Ugi reactions results from the adaptability of the so-called intermediate nitrilium ion, which has been trapped by various nucleophiles in addition to the carboxylic acid. In the Ugi-4CR, the carboxylic acid performs many important structural functions, encompassing activation of the intermediate imine, reversible addition to the nitrilium ion, and involvement in the irreversible Mumm rearrangement that forms the final diamide molecule (Figure 2). In just a handful of years, the Ugi group published a series of papers concerning the modification of the carboxylic acid input of the Ug-4CRs, such as cyanates, thiocyanates, azides, water, thioacetic acid, hydrogen selenide, and hydrogen sulfide, to produce diverse scaffolds, namely hydantoin imide scaffolds **6,** α-amino carboxamide **8,** α-aminoacylthioamides **11** (Scheme 1) [42]. Among them, the three essential parameters of diversity, novelty, and complexity are provided by the Ugi-4CRs, which provide convergent access to various tetrazole rings **7**. The bioisosterism of tetrazole scaffolds to carboxylic acid and amide moieties, as well as their metabolic stability and other advantageous physicochemical features, make them a leading family of heterocycles that are crucial to drug discovery and medicinal chemistry. Even though almost twenty FDA-approved medications contain 1H- or 2H-tetrazole substituents, little is known about their precise binding modes, structural biology, 3D conformations, or their general chemical behavior [43]. In the subsequent studies, El Kaïm et al. as a trailblazer in this field executed phenols and thiophenols as the acid component in the Ugi-4CR-entailing Smiles rearrangement to access *N*-aryl carboxamide **9** and α-arylamino thiocarboxamides **10** [44,45]. Phenols derivatives are more acidic and their acidity could be tuned by proper substitution onto the aromatic ring in comparison with aliphatic alcohols that are not acidic enough to react with isocyanides and imines [46].

In continuation of the breathtaking research mentioned above, natural and unnatural-amino acids, due to having both a primary amine and a carboxylic acid functional group, could be considered as privileged synthons for a three-component Ugi-reaction [47]. Ugi reaction of natural amino acids has meanwhile been deployed for structurally diverse polypeptoid as a direct and effective method [48].

**Scheme 2:**
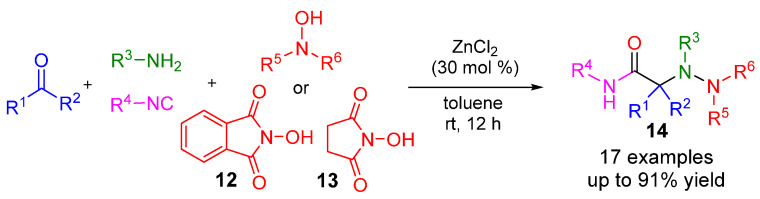
Using *N*-hydroxyimides as an acidic component in the Ugi-4CR.

As a new carboxylic acid isostere, the Ugi-4CR using *N*-hydroxyimides such as *N*-hydroxyphthalimides **12** and *N*-hydroxysuccinimides **13** has been described by Dömling and co-workers (Scheme 2) [49]. This process facilitates the synthesis of α-hydrazino amides **14**. This broad and facile reaction needs a catalytic amount of ZnCl_2_. Various amines, aldehydes, isocyanides, and *N*-hydroxyimides were used to produce compounds with moderate to good yields. This reaction demonstrates the creation of the N-N bond by cyclic imide migration in the Ugi reaction. Hence, the addition of *N*-hydroxyimide as a novel acid component in the Ugi reaction increases the variety of scaffolds.

**Scheme 3:**
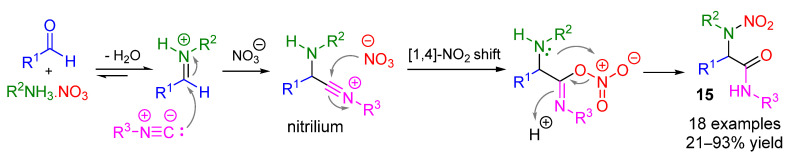
Using nitric acid instead of carboxylic acid in the Ugi-4CR.

In another alternative reactant study, the powerful nitric acid was used as carboxylic acids surrogates in the Ugi reaction. The use of nitric acid to catalyze the Passerini reactions and the use of water as the final nucleophilic trapping agent was previously reported in the literate only once. Utilizing a stoichiometric amount of acid without using water provides effective trapping of the intermediate nitrilium by the moderately nucleophilic nitrate anion, accompanied by intramolecular nitration. Authors may offer a feasible approach involving the intermolecular trapping of nitrilium by the nitrate anion by similarity with the mechanism of the conventional Ugi reaction [50]. As indicated in Scheme 3, the resultant nitro imidate might undergo a [1,4]-shift of the nitro group through a Mumm-type transfer to produce the equivalent nitramine **15**.

**Scheme 4:**
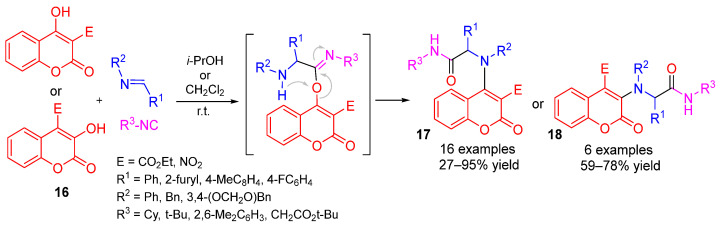
Hydroxycoumarins as an acid component in the Ugi-4CR.

The coumarin ring is a preferred motif and a prevalent structural moiety in a number of clinically active chemicals with anti-HIV antioxidant, anticancer, and anti-inflammatory utilities. Electron-withdrawing groups at position-3 and position-4 of hydroxycoumarins **16** have proven useful as acidic components in Ugi-3CR with imines and isocyanides (Scheme 4) [51]. The reaction proceeds easily at ambient temperature without catalysis, yielding highly convergent 3- and 4-coumarin enamines **17**, **18**. The conjugate addition-elimination rearrangement on the initial adduct is essential to this reaction and leads irreversibly to the ensued product. In another elegant report, it has been demonstrated that squaric acid can be utilized as a component in the Ugi-4CR reaction by way of a straightforward and effective application to the one-pot direct access to the original squaric-based symmetrical compounds [52]. This was accomplished by making use of facile reaction setting and an easy purification method.

**Scheme 5:**
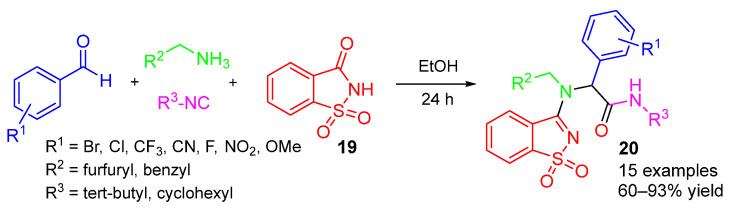
Saccharin as an acid component in the Ugi-4CR.

An interesting work using saccharin **19** as the acidic partner in the Ugi reaction was reported for the first time. It allows access to α-amino amides including imino saccharin **20** moiety from commercially available reactants, targeting structural variety and complexity (Scheme 5) [53]. The yield of the final product was reduced when acetonitrile, tetrahydrofuran, or dichloromethane were used as the reaction solvent in place of ethanol or methanol, while the maximum ethanol yield of the resultant product was 60% due to the stability of the iminium ion intermediate in polar protic media. Consequently, ethanol was utilized as a green medium for subsequent processes. The method has various benefits, such as gentle reaction conditions, a straightforward technique and facile workup, the absence of a catalyst, and appropriate yields.

There is a potential use of CH-acids in Ugi-type reactions. For instance, tetramic acids have sussesfully been deployed in an isocyanide-based MCRs for synthesis of valuable heterocyclic compounds [54,55]. In this vein, Meldrum’s acid as privileged CH acid has meanwhile been applied as a surrogate to carboxylic acid in a Ugi-type reaction. This pseudo five components reaction was serendipitously discovered whereby Meldrum’s acid and amines (two equivalent), aldehydes and isocyanides lead to triamide derivatives. This MCR based on Meldrum’s acid has opened a new avenue to single-pot direct preparation of an extended range of pharmaceutically and industrially outstanding heterocyclic and acyclic organic scaffolds [56]. In 2020, Matloubi Moghaddam, Goudarzi and co-workers developed a method for effective preparation of novel iminopyrrolidines through a four-component Ugi-type reaction of in situ generated dithiocarbamic acids from secondary amines and carbon disulfide with isocyanides and gem-dicyano olefins in the absence of any catalyst under ultrasonic conditions [57]. The authors, for the first time, reported a Mumm-type rearrangement involving dithiocarbamates, followed by intramolecular cyclization, which prepared the essential pyrrolidine structure. In an interesting report, Zhu and co-workers reported a five-component reaction for the synthesis of hexasubstituted benzenes using 2-isocyano-1-morpholino-3-phenylpropan-1-one that plays dual roles as an isocyanide and carboxilic acid in this Ugi-type reaction [58].

**Scheme 6:**
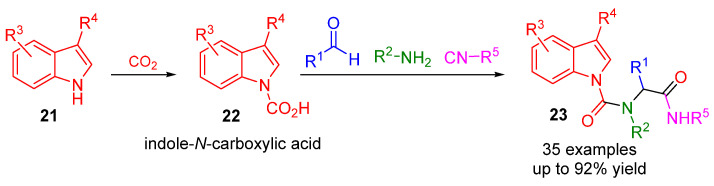
Preparation of indole carboxamide amino amides via pseudo Ugi-5CR.

Furthermore, the re-engineering logic enabled the transformation of non-elementary reactions with two or more components into higher-order MCRs. Ugi realized that carboxylic acids were able to be produced directly by the reversible reaction of CO_2_ with alcohols and then serve as the carboxylic acid’s component of Ugi MCRs. The conclusion portrayed with jubilation the notion of enhancing the dimensionality of MCRs. In 2019, a five-component Ugi reaction was efficiently devised for the preparation of indole carboxamide amino amides **23** from amines, aldehydes, isocyanides, and indole-*N*-carboxylic acids **22**, which were easily synthesized from indoles **21** and CO_2_ (Scheme 6) [59]. This process renders a remarkable method for generating indole-tethered peptide units with outstanding structural variety and shortness. In addition, the method has widely accessible substrates, a straightforward operation, and a rich variety. To show scalability, a gram-scale reaction was done, and the products were transformable into novel indole derivatives. A wide range of aldehydes efficiently proceeded in this synthetic strategy, and privileged functional groups for instance methoxy, cyano, bromo, chloro, and fluoro were tolerated. The 2-Naphthyl aldehyde and heterocyclic aldehydes with the moieties of pyridine, indole, furan, and thiophene and, moreover, unsaturated cinnamaldehyde were applicable in this process to render the final products in appropriate yields. The aliphatic aldehydes encompassing 3-phenylpropanal and iso-butyraldehyde successfully engaged in this synthetic route.

## 3. Alternative Components for Amines

**Scheme 7:**
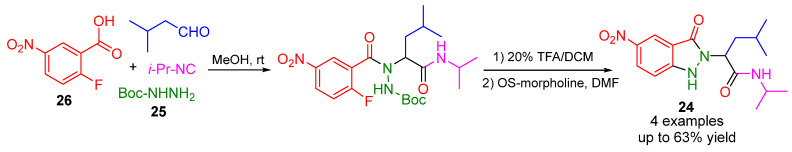
Boc-hydrazine as an amine component in the Ugi-4CR and post-Ugi transformation.

Aside from carboxylic acids, amines could also be replaced with alternative compounds to extend the complexity and skeletal diversity of the products for the Ugi MCRs. In this sense, Zinner et al. conducted a Ugi reaction with varying amounts of *N*-alkylated hydrazines and an excess amount of formaldehyde and cyclohexyl isocyanide [60]. Isocyanide was used to react with the iminium intermediate, leading to the final products. Additionally, the Krasavin research group has developed a novel class of hydrazinopeptide-like structures employing a variety of carboxylic acid hydrazide [61,62,63]. Hulme and Tempest have used a sophisticated variation of the hydrazo-Ugi process to make combinatorial libraries of compounds having a wide range of heterocyclic rings [64]. As can be seen in Scheme 7, a good yield of indazolones **24** was achieved by combining Boc-hydrazine **25** with 2-fluoro-5-nitrobenzoic acid **26** (along with a variety of aldehydes and isocyanides); this needed just two chemical phases and a product purification step. Following the Ugi phase, excess aldehyde (added to assure full production of the hydrazone intermediate) and unreacted acid were omitted by progressively applying polymer-supported tosyl hydrazine and diisopropylethylamine, respectively. After the Boc group was removed using trifluoroacetic acid, the SNAr-type cyclization proceeded effectively with the help of resin-bound morpholine to scavenge excess TFA and hydrofluoric acid. This synthetic strategy has paved the way to render novel useful heterocyclic compounds via post-Ugi transformation.

**Scheme 8:**
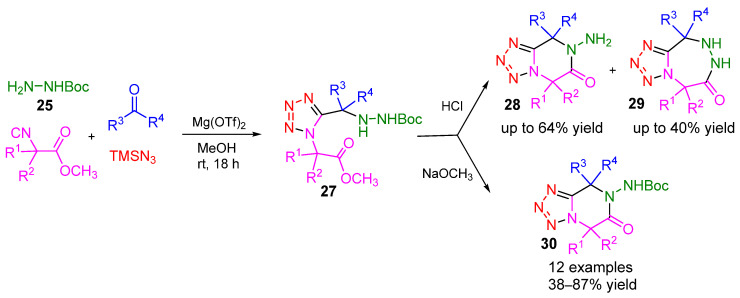
Boc-hydrazine as an amine alternative in the Ugi-4CRs for making three fused heterocycles by post-Ugi cyclization.

In a similar way, in 2016, Dömling et al. reported deploying *N*-Boc-protected hydrazine **25** in the Ugi tetrazole reaction to produce a series of highly substituted 5-(hydrazinomethyl)-1-methyl-1H-tetrazoles **27** via a single-pot, two-step reaction (Scheme 8). The final products were simply prepared with appropriate yield [65]. Next, the same research group developed a post-cyclization Ugi tetrazole adduct achieved by N-Boc protected hydrazine along with α-amino acid derived isocyanides under both acidic and basic conditions [66]. The 7-Aminotetrazolopyrazinone **28** and tetrazolotriazepinone **29** were obtained by a single-pot cyclization in acidic circumstances. In a basic environment, selective Boc-protected 7-aminotetrazolopyrazinone **30** products could be afforded by post-cyclization, with yields ranging from 38−87%.

**Scheme 9:**
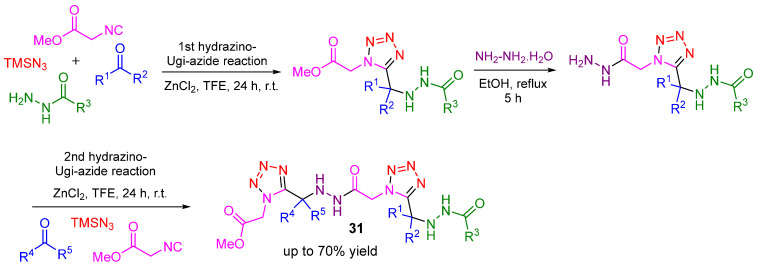
Preparation of acylhydrazines bearing 1,5-disubstituted tetrazoles.

In 2017, Barreto and co-workers reported an efficient strategy to access a wide spectrum of acylhydrazines bearing 1,5-disubstituted tetrazoles **31** in just three stages [67]. This synthetic route required merely the following stages: first, a four-component, single-pot hydrazino-Ugi-azide reaction; second, a hydrazinolysis; and third, an additional hydrazino-Ugi-azide reaction. The method offers a variety of benefits, including a straightforward synthetic technique with a high atom economy, an easy work-up, and also rapid generation of highly functionalized molecules in a few steps (Scheme 9).

**Scheme 10:**
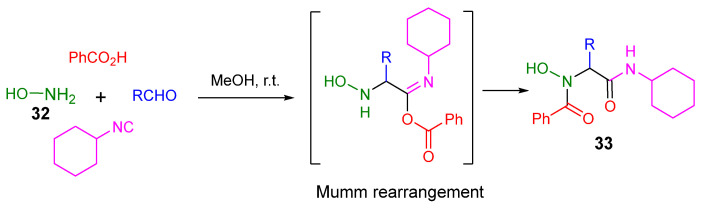
Hydroxylamine as an amine component in the Ugi-4CR.

Additionally, the Zinner group examined hydroxylamine as a potential alternative to amine in the Ugi reaction in 1969 [68]. The Ugi adducts with cyclohexyl isocyanide and different types of aliphatic and aromatic aldehydes can be produced in this reaction with fair to excellent yields by *N,O*-dimethylhydroxylamine, which was initially demonstrated to be capable of reacting in this reaction similarly to common amines. Interestingly, carboxylic acid, a main component of the Ugi reaction, was left out in this instance. Water effectively acted as the isocyanide-intercepting nucleophile in the company of an equivalent amount of hydrochloric acid. Going ahead, Moderhack et al. also implemented hydroxylamine **32** in Ugi four-component reaction, deploying benzoic acid as the acid component (Scheme 10) [69]. This study found that the only method used to rearrange the intermediate was N–O acyl migration, which produced the “internal” hydroxamic acids **33** in low yield.

**Scheme 11:**
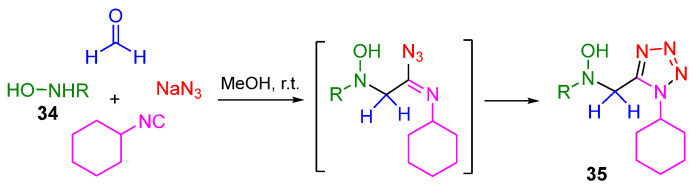
Ugi tetrazole reactions by hydroxylamine in place of common amines.

Shown in Scheme 11 is a pioneering example of applying hydroxylamine Ugi tetrazole reactions reported by the same research group. Combining hydroxylamine with sodium azide in place of carboxylic acid significantly broadens its application in the Ugi reaction [70]. Good to excellent yields of tetrazoles scaffolds **35** were obtained from the reaction with *N*-alkylhydroxylamines **34**, which apparently proceeded through an intramolecular dipolar cycloaddition of intermediate. However, the yields of these dimeric adducts from unsubstituted hydroxylamine are quite low.

**Scheme 12:**
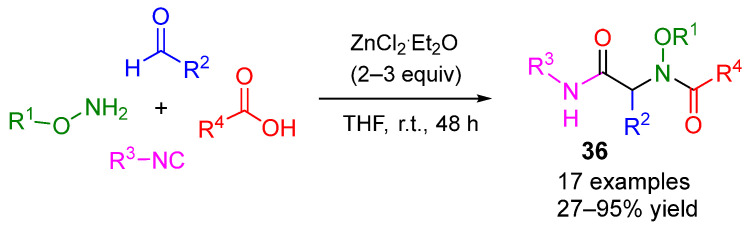
Zn-catalyzed Ugi-4CR using hydroxylamine as an amine component.

Approximately 30 years later, Basso et al. demonstrated that the poor reactivity of the *O*-protected oximes of aliphatic aldehydes towards isocyanides, prompting a reevaluation of the implement of hydroxylamine inputs in the Ugi reaction. In this report, when ZnCl_2_ was included in the reaction, appropriate yields of the desired O-protected hydroxamic acid derivatives **36** were achieved (Scheme 12) [71]. The optimized procedure (2–3 equivalent of the Lewis acids and the implement of an oxime in the reaction) was determined to be appropriate to a variety of aliphatic aldehydes and carboxylic acids, and it provided superior results in comparison with other promoters such as MgBr_2_, BF_3_, and TiCl_4_. Contrary to expectations, the authors did not observe the same reaction when presented with aromatic aldehyde oximes.

The same research group expanded this transformation to *N*-benzylhydroxylamine, a reagent capable of generating nitrones with carbonyl compounds that should be more reactive towards isocyanides than corresponding oximes [72]. In fact, the simple mixing of *N*-benzylhydroxylamine with an aliphatic aldehyde or ketone, an isocyanide, and a carboxylic acid in methanol produced highly acceptable quantities of α-acyloxyamino acetamides after two days at ambient temperature. More importantly, in this instance, the-adduct rearrangement can happen only via O→O acyl migration. 

**Scheme 13:**
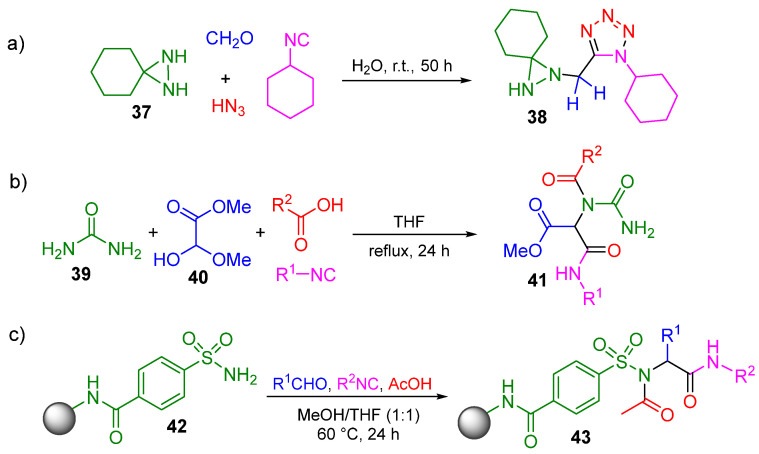
Diaziridine, urea, and sulfonamide as amine components in the Ugi-4CR.

During the preliminary research of Zinner and Bock, they discovered that diaziridine **37** as an amine component could create a low yield of the diaziridine tetrazole derivative **38** by reacting with formaldehyde, hydrazoic acid, and cyclohexyl isocyanide (Scheme 13a) [73]. Thereafter, as an intriguing example, urea has been employed as an amine component in the Ugi four-component reaction, in which an unidentified combination of an isocyanide and a carboxylic acid, urea **39**, and glyoxylic acid hemiacetal **40** produced an appropriate yield of *N*-aminocarbonyl amide **41** (Scheme 13b) [74]. In light of the commonly known mechanism of the reaction, the simple creation of this product is, in the opinion of the authors, challenging to explain. Likewise, it would be unexpected, when understanding the mechanism of the Ugi reaction, for primary sulfonamides, which are weak *N*-nucleophiles, to react as viable substitutes instead of amine component in the Ugi process. Meanwhile, *p*-toluenesulfonamide was illustrated to be approximately entirely inert towards carbonyl compounds, an acetic acid, and isocyanide in mixture of methanol and THF. By the time a Rink resin-bound benzenesulfonamide **42** was reacted with isocyanides, acetic acid, and an excess of aldehydes in a mix of THF-methanol, a variety of monoacetylated resin-bound products **43** was produced (Scheme 13c) [75]. Products of this phase were deacetylated using an aqueous methylamine solution (in THF), and then exposed to TFA cleavage, resulting in good yields of the Ugi adducts.

**Scheme 14:**
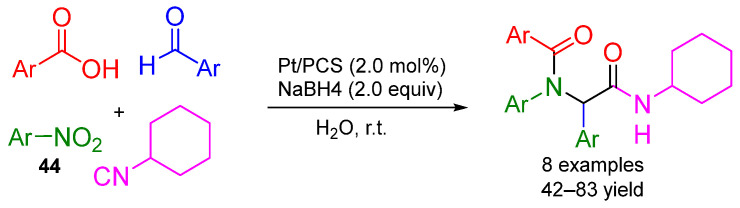
Nitrobenzene as an amine source for nitro group reduction in the Ugi reaction.

Further endeavors in this regard could open up an outstanding way for using new alternative compounds instead of amines. In 2020, on the basis of the Pt nanoparticles (NPs) on proline-functionalized cross-linked chitosan particles (Pt\PCS) that were precisely characterized, a novel heterogeneous Pt nanocatalyst was prepared [76]. Together with NaBH_4_, this catalyst developed a novel heterogenous catalyst for hydrogenation of nitrobenzene derivatives in water at ambient temperature. Furthermore, this catalytic system directly catalyzed the tandem Ugi-4CR with nitrobenzene derivatives **44**, in which nitrobenzene was transformed in situ into aniline, and subsequently consumed in the Ugi-4CR (Scheme 14). This catalytic approach was reusable and could be utilized multiple times without considerable activity loss.

## 4. Alternative Components for Aldehydes

**Scheme 15:**
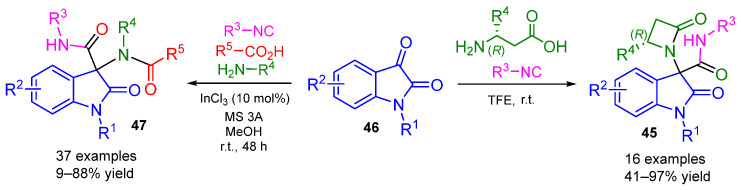
Isatin as a carbonyl component in the Ugi-4CR.

It is amply clear that Ugi reaction is swiftly carried out in the presence of aldehyde functional group as a carbonyl source and there are a battery of reports in this regard. Additionally, the ketone compound, meanwhile, could be successfully applied in this transformation. In order to increase the complexity of the Ugi adducts, some alternative substrates deployed as carbonyl sources are introduced. In this area, novel β-lactam-containing 3,3-disubstituted oxindole derivatives **45** have successfully been synthesized utilizing the Ugi four-centre, three-component reaction with isatin **46** as the carbonyl reagent (Scheme 15) [77]. Isatin is a valuable building block in medicinal chemistry because oxindole scaffolds illustrate an extensive range of biological activities, inclusively existing in various FDA-approved drugs, for instance, ziprasidone and ropinirole [78]. Utilizing chiral, non-racemic β-amino acids as bifunctional components or β-alanine, the reaction conditions were investigated and approved for a wide range of isatins and isocyanides containing chiral, non-racemic α-amino acids without using any catalyst. On the basis of their calculated physicochemical properties, final products have the potential to be drug-like and, consequently, to be utilized in drug discovery goals. Following previous reaction, isatin is applied as a privileged structure for the Ugi 4-center 3-component reaction to access oxindole-β-lactam as well as oxindole-*γ*-lactam hybrids. In order to investigate the inhabitation product’s potential, the final products were evaluated to inhibit relevant central nervous system targets, such as monoamine oxidases and cholinesterases. In addition, a drug-likeness evaluation was conducted, and a number of compounds demonstrate significant potential as selective butyrylcholinesterase inhibitors at low micromolar concentrations, with an intriguing predictive pharmacokinetic profile [79].

Another precursor isatin-based Ugi component reaction was reported by Pineiro et al. in 2021 for the synthesis of oxindole derivatives **47** catalyzed by indium(III) trichloride [80]. Different *N*-substituted and *N*-unsubstituted isatins encompassing both electron-donating and -withdrawing groups on the phenyl ring were found to react successfully using this synthetic approach. Most of the resulting compounds exhibited modest and sub-micromolar range antiproliferative activity against six different tumor cell lines, with the most active molecule reaching nanomolar activity.

## 5. Alternative Components for Isocyanides

Isocyanides, symbolically speaking, have been likened to “Jekyll and Hyde”, or a person with bipolar disorder, since they allow for highly efficient scaffold construction, which in turn facilitates hit-tolead efforts, yet they are also frequently utterly odorous to the senses. Scientists recently observed coming on board with “isocyanide phobia” owing to the strong odors; nevertheless, this quickly transforms into “isocyanide mania” once the full depth of the productivity increases and the inherent exploratory capacity of IMCRs is revealed [81,82,83,84,85]. Nowadays, isocyanides are considered as a peculiar functional organic group that undergo remarkable chemical reactions due to their exceptional reactivity for electro- and nucleophiles adding onto the isocyanide carbon [86,87]. In light of their (incorrect) association with toxicity and the extremely odorous scent of a certain number of isocyanide derivatives, the production of isocyanides presents the biggest challenge when it comes to isocyanide-based MCRs. Considering the indisputable importance of isocyanides, introducing new and expeditious methods for in situ generation of isocyanides can be taken into account as an extraordinary remedy for Ugi reactions as it eliminates the need to isolate the stinky isocyanides and shortens the time to the desired product without compromising scaffold and structural diversity. Generally, this in situ method represents a significant step forward in the quickly developing isocyanide chemistry area and in IMCR in particular [88].

**Scheme 16:**
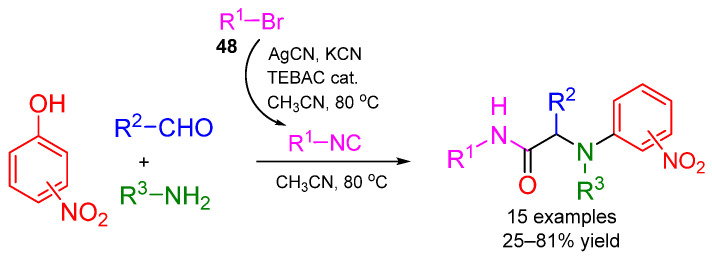
Benzyl bromide as an isocyanide precursor in the Ugi-4CR.

At first, El Kaïm’s group embarked on deploying in situ generated isocyanide approach for Ugi reactions. They focus on Ugi–Smiles and conventional Ugi reactions with in situ generated isocyanides, which are synthesized via the reaction of benzyl bromide **48** derivatives with AgCN and KCN in the company of a phase-transfer catalyst, considered a significant advancement in this area of study (Scheme 16) [89,90]. This sequence ignores isolating volatile and foul-smelling intermediates, culminating in comparable yields of the desired adduct compared to conventional conditions. This approach has paved the way for new synthetic strategies, and, in addition, several viable methods for producing and collecting isocyanides on-site have been presented.

In a breakthrough achievement, Sharma and co-workers devised an automated continuous microfluidic system for in situ preparation of isocyanides and their conversion into ensued products with minimal environmental impact. To alternate carbonyl and carboxylic acid components, the authors prepared pyrrolidin-2-ones by substituting levulinic acid (a natural product derived from plant biomass). These transformations are carried out by passing the substrates via perfluoroalkoxy tubing held in an ultrasonication bath and mixing in a T-mixer. N-substituted formamide in the presence of POCl_3_ accompanied by DIPEA as a base in toluene turns into isocyanides derivatives. Then, Ugi adducts were produced with good-to-excellent yields (up to 88%) through reaction of isocyanides with levulinic acid, as well as amines in DMF. A residence period of 24.5 min under ultrasound-promoted conditions at ambient temperature was determined to be the optimal condition for executing the continuous Ugi reaction [91].

**Scheme 17:**
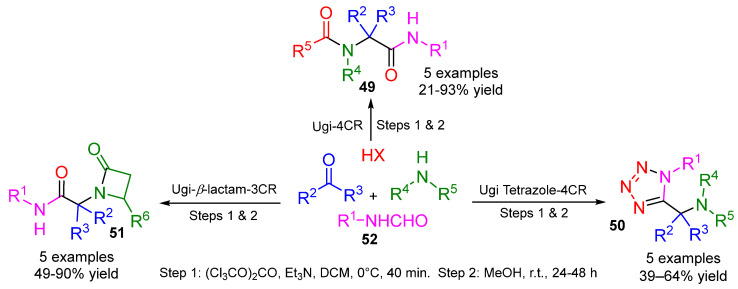
Ugi MCRs using in situ prepared isocyanides from formamides.

In this context, the Dömling research group’s study is one that provides a ripe avenue and deserves special note in relation to the isocyanide-free Ugi MCRs [92]. They harnessed the classical Ugi-4CR **49**, Ugi tetrazole reactions **50** via isocyanide-less IMCR, and also demonstrated the direct synthesis of β-lactams **51** through a three-component reaction (Scheme 17). Isocyanides were synthesized by treating the formamides derivatives **52** with Et_3_N as a base and in the company of triphosgene as a dehydrating agent. As for tetrazoles, these in situ prepared isocyanides were reacted with aldehydes, benzyl amines, and trimethylsilyl azide in MeOH in the same pot to yield the 1,5-disubstituted tetrazoles **50** with moderate-to-good yield. These types of isocyanides also reacted in situ with aldehydes and β-amino acids in MeOH at ambient temperature to obtain ensured β-lactams **51**. In addition, using model examples, they revealed that the in situ preparation of isocyanides is advantageous for obtaining higher yields of β-lactams in comparison with the conventional procedure. This outcome emanates from enhanced solubility of the β-amino acid because of its salt effect. It was determined that the yields and reactant range were equivalent to, or better than, the original processes employing isolated isocyanides.

**Scheme 18:**
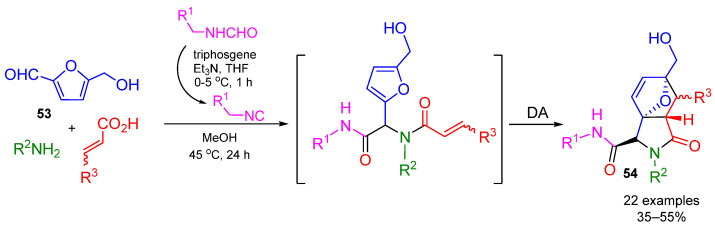
Formamides in Ugi-4CR followed by an intramolecular Diels–Alder reaction.

The same research group explored the synthesis of tetrasubstituted imidazole, disubstituted benzimidazole, and substituted 1,4-benzodiazepine via post-modification of Ugi reaction adduct, achieved deploying isocyanide generated in situ by the Leuckart–Wallach synthetic route [93]. Moreover, to expand the repertoire of novel chemical compounds using isocyanide-less IMCR, in 2019, Golubev and co-workers devised the tandem reaction of an in situ version of the Ugi-4CR, and then intramolecular Diels–Alder reaction, deploying 5-hydroxymethylfurfural **53** as one of the substrates for a straightforward single-pot, three-step, synthesis of hydroxymethyl-substituted epoxyisoindolones **54** (Scheme 18) [94]. Ensured products were generated with appropriate yields, and more importantly, most of them were achieved as a single diastereomer.

## 6. Conclusions 

The Ugi-4CR is a topic of worthwhile importance in the MCRs that have aroused interest among organic and medicinal chemists. A variety of pseudopeptides, druggable molecules, natural compounds, heterocycle scaffolds, macrocycles, and functional material could be produced by the Ugi-4CRs. This important development in isocyanide-based MCR uses an amine, an aldehyde or ketone, an isocyanide, and a carboxylic acid to create derivatives of *a*-acetamido carboxamide. In a pot, atom, and step economic transformation known as the Ugi reaction, four diverse starting materials couple to create a wide range of complex molecules. Aside from the original carboxylic acid, other nucleophiles such as azide, *N*-hydroxyphthalimide, thiols, saccharin, phenol, thioacetic acid, water, hydrogen sulfide, squaric acid and others have trapped out the so-called intermediate nitrilium ion, allowing for a myriad of Ugi reactions to take place. Additionally, there are several alternative reagents for amines such as hydroxyl amin, hydrazine, nitrobenzene, and urea, among others, which lead to more complex organic scaffolds. Recently, deploying isatins as carbonyl component and various new surrogates instead of isocyanides such as formamide and benzyl bromide make Ugi reaction an aptly diversity-oriented reaction. We anticipate that by reviewing this topic, organic chemists will better understand the versatility and synthetic potential of Ugi-4CRs and their novel modification. Experts will be able to provide new alternate components for Ugi-4CRs to create novel structures that might be used as medicinal chemistry medication candidates. We believe that, as predicted by I. Ugi and other luminaries of contemporary MCR-based organic synthesis, the Ugi-4CRs and all of its derivatives will be employed more and more to continue filling the chemical space in a robust and appealing manner.
